# Susac Syndrome Following COVID-19 Vaccination: A Case Report

**DOI:** 10.3390/vaccines10030363

**Published:** 2022-02-25

**Authors:** Po-Jui Chen, Yi-Sheng Chang, Chen-Chee Lim, Yu-Kuei Lee

**Affiliations:** 1 Department of Ophthalmology, National Cheng Kung University Hospital, College of Medicine, National Cheng Kung University, Tainan 704, Taiwan; bread358@gmail.com (P.-J.C.); willis@mail.ncku.edu.tw (Y.-S.C.); 2 Department of Ophthalmology, College of Medicine, National Cheng Kung University, Tainan 701, Taiwan; 3 Department of Ophthalmology, Tainan Hospital, Ministry of Health and Welfare, Tainan 700, Taiwan; internj91@gmail.com

**Keywords:** Susac syndrome, COVID-19 vaccine, adenoviral vector vaccine, case report

## Abstract

Due to the COVID-19 pandemic, numerous vaccines have been developed for the disease. However, with large-scale vaccination has come the gradual emergence of immunological phenomena caused by these new vaccines. Herein, we report a 48-year-old female with a sudden onset of inferior visual field defects in the left eye following her first dose of the ChAdOx1 vaccine. Dilated fundus examination combined with optical coherence tomography and fluorescein angiography confirmed the diagnosis of branch retinal artery occlusion. Within 4 weeks following vaccination, symptoms associated with hearing impairment developed, and magnetic resonance imaging revealed leptomeningeal enhancement. The diagnosis of Susac syndrome (SS) was confirmed. The development of SS may be caused by endotheliopathy resulting from the molecular mimicry of the ChAdOx1 vaccine. Clinicians should be aware of the symptoms of SS, which may develop after COVID-19 vaccination. Further experimental surveillance and case–control studies are required to confirm this relationship.

## 1. Introduction

The coronavirus disease 2019 (COVID-19) pandemic, caused by the novel severe acute respiratory syndrome coronavirus 2 (SARS-CoV-2), has affected over 260 million people and caused over 5.1 million deaths globally [1]. Vaccination has proven to be an effective method to control the spread of COVID-19 and, currently, several vaccines and candidates are approved and tested by different countries worldwide [2]. To date, 7.85 billion doses of COVID-19 vaccines have been administered globally and have resulted in one of the largest-scale vaccination campaigns in modern history [3]. Ocular adverse events, including abnormalities in anterior and posterior segments, also present in some cases after COVID-19 vaccination, and retinal disease is one of the most common adverse events [4]. With the unprecedented scale of vaccination for COVID-19 development, the number of rare adverse events is increasing. Most are mild and transient, eventually resolving, but some of them are rare and severe, raising safety concerns regarding vaccines. Herein, we report a case that initially presented as ophthalmological abnormalities following COVID-19 vaccination and which was eventually diagnosed as Susac syndrome (SS).

## 2. Case Presentation

**Figure 1:**
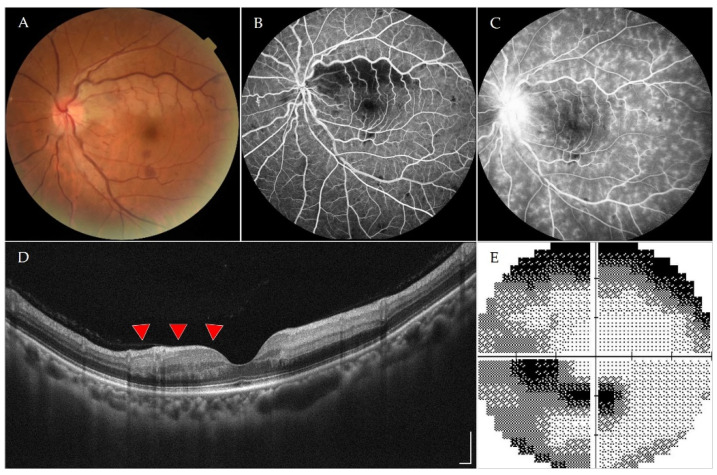
(**A**) Color fundus imaging of the left eye revealed a pale retinal patch around the superior arcade. (**B**) Fluorescein angiography of the left eye showed delayed superior branch retinal artery filling. (**C**) Fluorescein angiography of the left eye in the late phase showed disc leakage and fluffy vessels. (**D**) Optical coherence tomography imaging of the left eye revealed retinal edema at the superior area of the macula (red arrows). Scale bar equals 250 μm. (**E**) Visual field testing of the left eye revealed inferior arcade scotoma and peripheral visual field defect.

A 48-year-old, previously healthy female with no comorbidities or drug allergies came to the ophthalmology service due to a sudden onset defect of the inferior visual field in her left eye for 1 day without redness or pain. The patient had received her first dose of the ChAdOx1 nCoV-19 vaccine (AstraZeneca) 4 weeks ago. Apart from a fever up to 39 degrees Celsius for three days and swelling at the injection site lasting approximately two days after vaccination, she had no other side effects. She also denied having any past ocular disease or use of medication, and there was no contributory family history previously reported. On examination, the best corrected visual acuity was 20/20 in both eyes, and intraocular pressures were 19 and 20 mmHg in her right and left eyes, respectively. The slit-lamp biomicroscope showed normal anterior segment findings, and a dilated fundus examination revealed a pale retinal patch around the superior arcade in her left eye (Figure 1A). On fluorescein angiography, delayed branch retinal artery filling was observed in her left eye, and disc leakage with fluffy vessels was also noted in the late phase (Figure 1B,C). Optical coherence tomography imaging of her left eye showed retinal edema at the superior area of the macula (Figure 1D). Visual field testing of her left eye revealed inferior arcade scotoma and peripheral visual field defects (Figure 1E). Therefore, she was diagnosed with branch retinal artery occlusion (BRAO) with vasculitis of the left eye.

The laboratory examination revealed only a slightly elevated erythrocyte sedimentation rate (20 mm/h) and hyperlipidemia. Electrocardiography, carotid ultrasonography, ophthalmic artery ultrasonography, and echocardiography were all within normal limits. There was no thrombocytopenia, and the fibrin/fibrinogen degradation products were normal. The patient had no abdominal pain, dyspnea, or focal neurological deficit.

**Figure 2:**
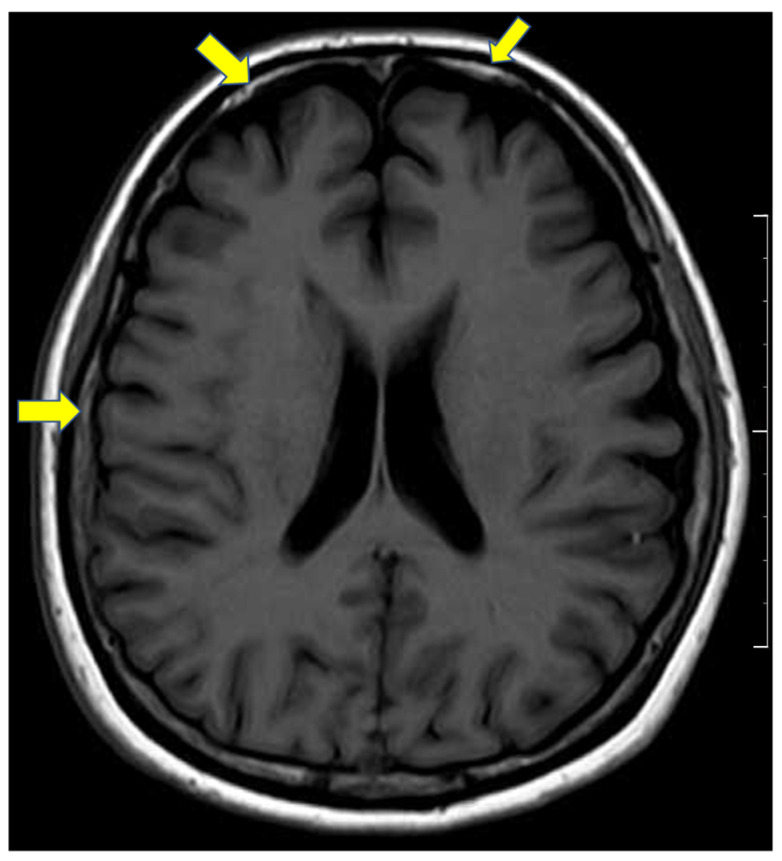
Magnetic resonance imaging (T1 series) showed continuous leptomeningeal enhancement (yellow arrows). Scale bar equals 10 cm.

Magnetic resonance imaging (MRI) scans 3 weeks after the initial visit showed continuous leptomeningeal enhancement (Figure 2). Without the presentation of headache, cognitive dysfunction, gait disturbance, and dysarthria, hearing impairment with intermittent tinnitus developed one month after the initial presentation of ocular symptoms, and the pure tone average test revealed mild hearing loss at 8000 Hz in her left ear. No specific finding was recognized by neurological examination.

Due to the concurrence of leptomeningeal enhancement upon brain MRI, BRAO, and hearing loss, the diagnosis of SS was confirmed. We prescribed brimonidine tartrate ophthalmic solution 0.15% (ALPHAGAN^®^ P, Allergan, Waco, TX, USA) twice daily for the left eye due to BRAO. Three months after the initial visit, the best-corrected visual acuity was 20/20 in both eyes, and there was no progression of visual field defects. Given the relatively mild involvement and stability of vision and hearing without other focal neurological signs, there is currently no systemic treatment. Repeated brain MRI and pure tone average tests were arranged regularly. Steroids or intravenous immunoglobulins may be used as immunosuppressive treatment if any symptoms or signs progress.

## 3. Discussion

SS is a rare disease that has an annual incidence of 0.024/100,000 and which is characterized by the triad of encephalopathy, branch retinal artery occlusions (BRAOs), and hearing loss [5,6]. The ocular symptoms of SS often present as blurred vision or deficits of the visual field, and the most common finding is BRAO. The pathology of SS mostly remains unknown, but it is presumed to be mediated through CD8+ autoimmune endotheliopathy, which leads to microangiopathy in the retina, cochlea, vestibular labyrinth, and brain [7]. The initial symptom can be loss of vision alone or combined with hearing loss. There are still no standard protocols for diagnosis and treatment due to the rarity of the disease and diversity in the clinical course [8]. The diagnosis of SS is based on fundus fluorescein angiography, brain MRI, and audiometry in cases of clinical suspicion. Reviewing past literature revealed no concrete evidence of a vaccine inducing SS. However, several case reports had claimed the possible relationship between SS and different vaccines. Landa et al. presented one middle-aged male developing into suspected SS 10 days after smallpox vaccination [9]. Recently, Silva et al. reported several vascular retinal disorders after COVID-19 vaccines, which included two cases with a highly possible diagnosis of SS 14–15 days after CoronaVac (Sinovac) [10]. In our present case, the SS was diagnosed based on clinical symptoms and imaging evidence, which showed highly suspicious SS that may have been induced by the ChAdOx1 vaccine (AstraZeneca).

The novel ChAdOx1 vaccine, an adenoviral vector vaccine using a replication-incompetent adenovirus as a vector to deliver the DNA signal sequence of the spike protein, is one of the most commonly used COVID-19 vaccines worldwide [11]. Up to 94.6% of people receiving the ChAdOx1 vaccine reported at least one side effect [12]. The common local side effects were injection site pain, swelling, and redness. The common systemic side effects, including fatigue, muscle pain, chills, feeling unwell, nausea, and headache, were mostly resolved within 1–3 days. According to the safety research, the incidence of side effects of the ChAdOx1 vaccine were higher in females and previously infected participants. Interestingly, chronic illnesses and medical treatments did not increase side effect incidence and frequency [12]. In this present case, a generally healthy woman presented with fever, chills, and injection site swelling as the initial side effects following the vaccination, and there were resolved within three days. On the other hand, studies have reported that some clinical features from the syndromes of vaccine-induced immune thrombocytopenia and thrombosis (VITT), which had high mortality, were associated with the ChAdOx1 vaccine [13]. Antibodies stimulated by platelet factor 4 (PF4) bind to platelets after the injection of the adenoviral vector vaccine, activating the coagulation system of platelets, and resulting in clinically significant thromboembolic complications. Anti-PF4 antibodies also activate monocytes, neutrophils, and endothelial cells, demonstrating the characteristic pancellular activation by the anti-PF4 antibodies [14]. The pancellular activation by anti-PF4 antibodies makes them a contributing or adding factors for SS. Given the extensive immune reaction of anti-PF4 antibodies induced by the ChAdOx1 vaccine, SS, which is possibly linked to autoimmune endotheliopathy, should be considered as one of the possible complications following vaccination.

The immunological response to the spike antigen or to components of the chimpanzee or human adenovirus can cause ocular diseases [4]. Recently, several systemic reviews of the COVID-19 vaccine have demonstrated multiple ocular manifestations following vaccination with the ChAdOx1 vaccine, which included corneal graft rejection, acute macular neuroretinopathy (AMN), multifocal choroiditis, acute retinal necrosis, and optic neuritis with longitudinal extensive transverse myelitis [4,15]. Superior ophthalmic vein thrombosis and cerebral venous sinus thrombosis were also reported following the ChAdOx1 vaccine, which may be also related to the mechanism of VITT. After ChAdOx1 vaccination, soluble spike variants that can be accidentally generated during transcription may induce thrombotic events via an antibody-mediated mechanism when binding to ACE2-expressing endothelial cells in blood vessels [16,17]. The molecular mimicry effect causes retinal pathologies by creating antibodies against self-antigens and inducing autoimmunity via the adaptive immune system [18]. Moreover, the proinflammatory or procoagulant response toward vessel endothelial cells may also enhance and induce retinal pathologies [19]. In the ocular manifestations mentioned above, AMN, consisting of microvascular ischemia in the deep capillary plexus of the retina and central retinal vein occlusion, has been reported following COVID-19 vaccination [20,21,22,23,24,25,26,27]. Similarly, we believe that the branch retinal artery occlusion, in our case, may have been triggered by the vaccine-induced immune reaction.

A recent report also presented a case of SS development following SARS-CoV-2 infection [28]. The patient developed cognitive dysfunction with ischemic cortical strokes associated with leptomeningeal enhancement on MRI and retinal arteriolar vasculitis, but the sensorineural hearing was relatively spared. The spike protein of SARS-CoV-2 attaches to the angiotensin-converting enzyme 2 (ACE-2) receptor on endothelial cells, which may lead to endothelial cell dysfunction and trigger a cytokine cascade causing severe coagulopathies and occlusive vasculopathy [28]. SS may belong to the spectrum of post-COVID-19 inflammatory vasculopathies. The presence of the spike protein, either from SARS-CoV-2 or from adenoviral vector–based COVID-19 vaccines, may cause endotheliopathy and further vasculopathy and directly result in the autoimmune activation of SS.

In our case, the latency period between the vaccination and symptom onset of SS was 4 weeks, which is similar to the latency in the above-mentioned case of SS after infection with SARS-CoV-2 [28]. Moreover, consistent with our period, studies have reported that the onset gap of vaccine-induced immune thrombocytopenia from the ChAdOx1 vaccine is 5 to 48 days (median, 14 days), which reveals possible immune-related endotheliopathy with a latent presentation [13,29]. Finally, the development of SS has a relatively slow course. Previous studies have shown that it takes an average of 7 months to develop the full triad of SS symptoms after the initial symptoms [30]. Accordingly, we believed that the present case is the first reported case of SS following COVID-19 vaccination, and one of few rare diseases documented.

## 4. Conclusions

Our case highlights the possible association between SS and adenoviral vector-based COVID-19 vaccination. Clinicians should be aware of this possible complication if a patient presents symptoms correlated with SS after COVID-19 vaccination. However, further studies are required to confirm this relationship and possibly elucidate the pathogenesis of this phenomenon.

## Data Availability

The datasets used and/or analyzed in the course of the current study are available from the corresponding author on reasonable request.
